# Age effect analysis of different gender groups in spatial ability test based on virtual reality technology

**DOI:** 10.3389/fpsyg.2024.1494048

**Published:** 2025-01-22

**Authors:** Yangyang Guo, Mengdi Zhang, Jiangpeng Gu, Qiyang Liu, Xinyang Liu, Jie Wang, Fangfang Ma, Lihong Zhai, Jianlin Qi, Zhanguo Jin

**Affiliations:** ^1^Air Force Clinical College, Anhui Medical University, The Fifth School of Clinical Medicine, Anhui Medical University, Hefei, Anhui, China; ^2^Department of Otolaryngology, The Second Affiliated Hospital of Soochow University, Suzhou, Jiangsu, China; ^3^Hebei North University, Zhangjiakou, Hebei, China; ^4^The Department of Vertigo Medical Centre, Air Force Medical Center, Air Force Medical University, Beijing, China; ^5^The Department of Psychology, Air Force Medical Center, Air Force Medical University, Beijing, China

**Keywords:** spatial cognition, spatial visualization, spatial orientation, virtual reality, cognitive development

## Abstract

**Purpose:**

The objective of the present study was to examine the impact of age and cognitive autonomy across various gender categories. Moreover, this research seeks to delve into the dissociation of diverse spatial aptitude assessments, with the aim of elucidating the intricate mechanism underpinning spatial capability.

**Method:**

Based on virtual reality technology, this study conducted spatial ability tests on 312 volunteers, aged from 18 to 90 years old, including R-letter rotation test, S-M mental rotation, surface development test and maze test.

**Results:**

The analysis revealed that the spatial ability of men decreases with age, but the spatial ability of women between 28 and 37 years old is better than that of other age groups. Males outperformed females in most visual ability tests, but there was no significant difference in some age groups. There was no significant correlation between the R-letter rotation test and the S-M mental rotation test, and the two tests were independent. The relationship between visual ability and orientation ability is different in different spatial test indicators.

**Conclusion:**

This investigation further elucidates the dissimilarities in the age-related characteristics of spatial aptitude among diverse gender cohorts, as well as the autonomy of various spatial aptitude assessments. Such distinctions are instrumental in occupational preference for disparate groups, calling for comprehensive and meticulous inquiries into the maturation of spatial proficiency by researchers.

## Introduction

Spatial ability is an important core of the development of human thinking. From the establishment of the concept of object shape, size and distance to the representation of the physical world by the representation system, it is the key factor to determine how the individual perceives and interacts with the surrounding environment. The spatial test of college-aged men and women showed that men needed less time to solve mental conversion tasks than women, but there was no systematic attempt to test gender differences (Cooper, 1975; Langlois et al., 2024). The most important age-related change in cognitive function is cognitive decline (Rivera et al., 2021; Xu et al., 2023). However, some studies have shown that there is no significant correlation between spatial orientation ability and age (Daniel, 1987).

Evolutionary and cognitive theories point out the difference between spatial visualization ability and spatial orientation ability (Hegarty and Waller, 2005). Psychological studies have shown that these two abilities are affected by different sensory information and brain structure (Malanchini et al., 2020). However, other psychological studies have shown that spatial visualization and spatial orientation may be closely related (Borich and Bauman, 1972; Hegarty, 2024). For example, the theory of gender difference evolution suggests that individual differences in spatial visualization ability are the product of male and female under different pressures in the process of biological evolution (Jones et al., 2003). Studies have shown that there is a positive correlation between spatial visualization and spatial orientation (Cooper, 1975). However, Spatial orientation ability measured at the same time cannot fully account for spatial visualization ability, and vice versa (Daniel, 1987).

Visualization is the representation of objects relative to each other in space the ability to change and move mechanically (Linn and Petersen, 1985). Spatial orientation refers to the recognition and direction judgment in three-dimensional space, the speed and height of objects perception and control (Chirico et al., 2016). The spatial visualization test is less affected by factors in the real environment. However, the research on spatial orientation ability has been hindered. Spatial orientation test is a self-object representation system, which may be costly and inefficient in practice (Barratt, 1953). Until recent years, advances in virtual reality (VR) technology have provided a new tool for studying spatial orientation skills in real environments, and such differences between participants can be controlled and standardized. Research evaluating the effectiveness of navigation skills using VR measurement shows that VR technology can reflect the real navigation process (Coughlan et al., 2019; Hegarty et al., 2006).

Most studies focus on the method of distinguishing spatial ability tests, rather than examining commonalities and differences in a wide range of spatial abilities. There is no unified conclusion on the changes of spatial ability in different gender and age groups, the correlation of different spatial ability tests and the independence of different spatial ability. The research overcomes the limitations of the real environment and carries out spatial visualization ability and spatial orientation ability based on virtual reality technology. This study will comprehensively analyze the development trajectory of spatial ability in the life cycle of adult groups of different genders, explore the independence of different spatial ability tests, the independence of cognitive ability and the effects of age and gender, and provide more evidence on the practical significance of spatial tests in providing high-level spatial ability for occupations and patients with specific spatial ability disorders.

## Methods

A total of 312 participants participated in the spatial test based on virtual reality technology. In the analysis of the results, we only included qualified data. Because the test is based on virtual reality technology, in order to eliminate the noise caused by technical problems, tutorials and practice tests are set up. A total of 300 testers qualified in our data set. Of these participants, all 300 provided age information. We select one of the five age groups to report their age (starting at 18 years old, select the age group in a 10-year-old group window until age 58 or older). We merged the elderly and late adulthood into the over-58 age group because there were fewer participants in these two age groups. Informed consent was obtained from all participants prior to participating in the assessment.

In addition, in order to analyze the correlation between different spatial visual ability tests, a separate sample (*n* = 143, ≥18 years old) was subjected to visual tests (including S-M mental rotation test, R letter rotation test, surface development test).

### S-M mental rotation test

**Figure 1:**
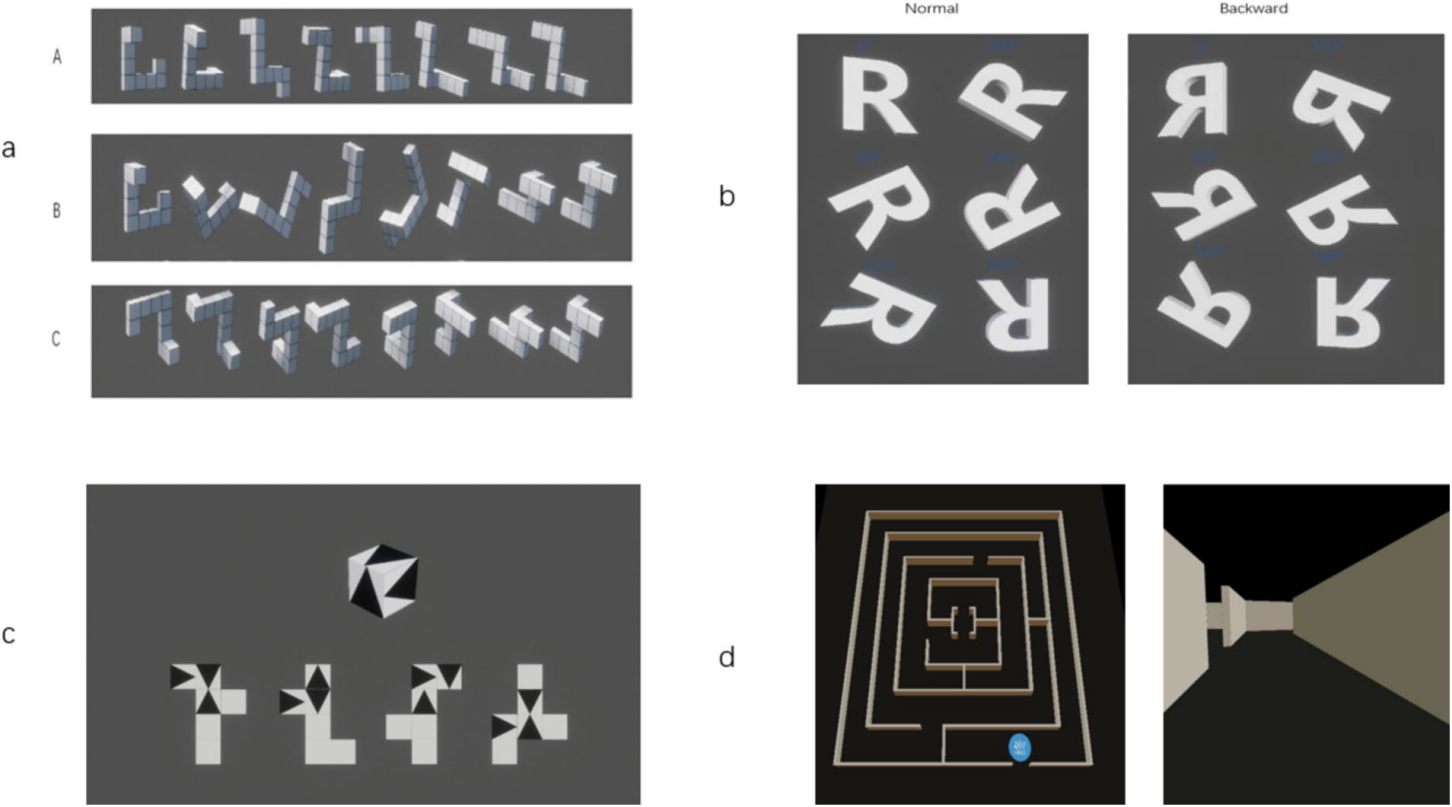
Experimental task. **(A)** Screenshots of mental rotation assessment based on virtual reality technology. Each time a pair of stimulus models are randomly presented, participants need to judge whether the test stimulus cube is the same as the original stimulus cube or the mirror relationship as soon as possible while ensuring the accuracy. **(B)** R-letter rotation evaluation screenshot based on virtual reality technology. Each time, a stimulus model is randomly presented. Participants judge whether the stimulus model is a positive image or a mirror image, and make judgments as soon as possible while maintaining high accuracy. **(C)** Screenshots of surface development assessment based on virtual reality technology. Each time a square three-dimensional cube and four unfolded planes are presented, participants determine which unfolded image can be restored to the shape of the cube in the title, and make a judgment as soon as possible while maintaining a high accuracy. **(D)** Screenshots of maze test evaluation based on virtual reality technology. The initial position of the subject is the starting point. In the process of travel, a small map can be adjusted by operating the handle. When the subject began to walk, the system began to time, recording the time spent from the starting point to the end point and the number of collisions.

The S-M mental rotation test is based on the Shepard-Metzler paradigm (Shepard and Metzler, 1971). We use HTC VIVE as an interactive experience platform to develop a three-dimensional visual spatial visualization ability test system for immersive experience (Figure 1A). In the test, a pair of stimulus models (the original stimulus cube is on the left side, and the test stimulus cube is on the right side) are randomly presented each time. Subjects are required to judge whether the test stimulus cube is the same relationship or the mirror relationship with the original stimulus cube, and to make judgments as soon as possible while maintaining high accuracy. There are 24 tests in total.

### R-letter mental rotation test

The R-letter mental rotation test is based on the Cooper paradigm (Cooperau and Shepard, 1973). The test uses an immersive experience spatial visualization test system (Figure 1B). In the test, a stimulus model is randomly presented each time, and the subjects are required to judge whether the stimulus model is a positive image relationship or a mirror image relationship, and to make a judgment as soon as possible while maintaining a high accuracy rate. The test character uses the 3D model of the uppercase letter R, and the R character is divided into six angles: 0°, 60°, 120°, 180°, 240°, and 300°, with a total of 12 questions.

### Surface development test

The surface development test is based on spatial ability simulation tests such as Newton (Newton and Bristoll, 1979). The three-dimensional visual modeling software (3DMAX) is used to present a three-dimensional cube composed of 1 cm × 1 cm square cardboard and four unfolded plans (Figure 1C). The spacing between the four plans is 0.5 cm. After folding the plan, a cube can be formed. The subjects (Slough et al., 2010) are required to perform psychological operations such as psychological folding and unfolding to determine which unfolded image can be restored to the shape of the cube in the question, a total of 4 questions.

### Maze test

According to the classic Wechsler Intelligence Scale for Children-Revised (WISC-R) maze subtest (Costa et al., 2018), we generated a three-dimensional maze in a virtual reality environment (Figure 1D). The initial position of the subject is the starting point. Before starting to walk, observe the panoramic map of the radar maze provided in the middle of the field of view, update the location information in real time and provide route navigation. When the subject starts walking, the system starts timing, which is used to record the time spent from the starting point to the end point. Collision detection is provided at each corner collision to indicate whether the front is the wrong route and record the number of errors. When the subjects are walking, they can pull out a small map to judge their location information and path clues in real time.

## Results

**Figure 2:**
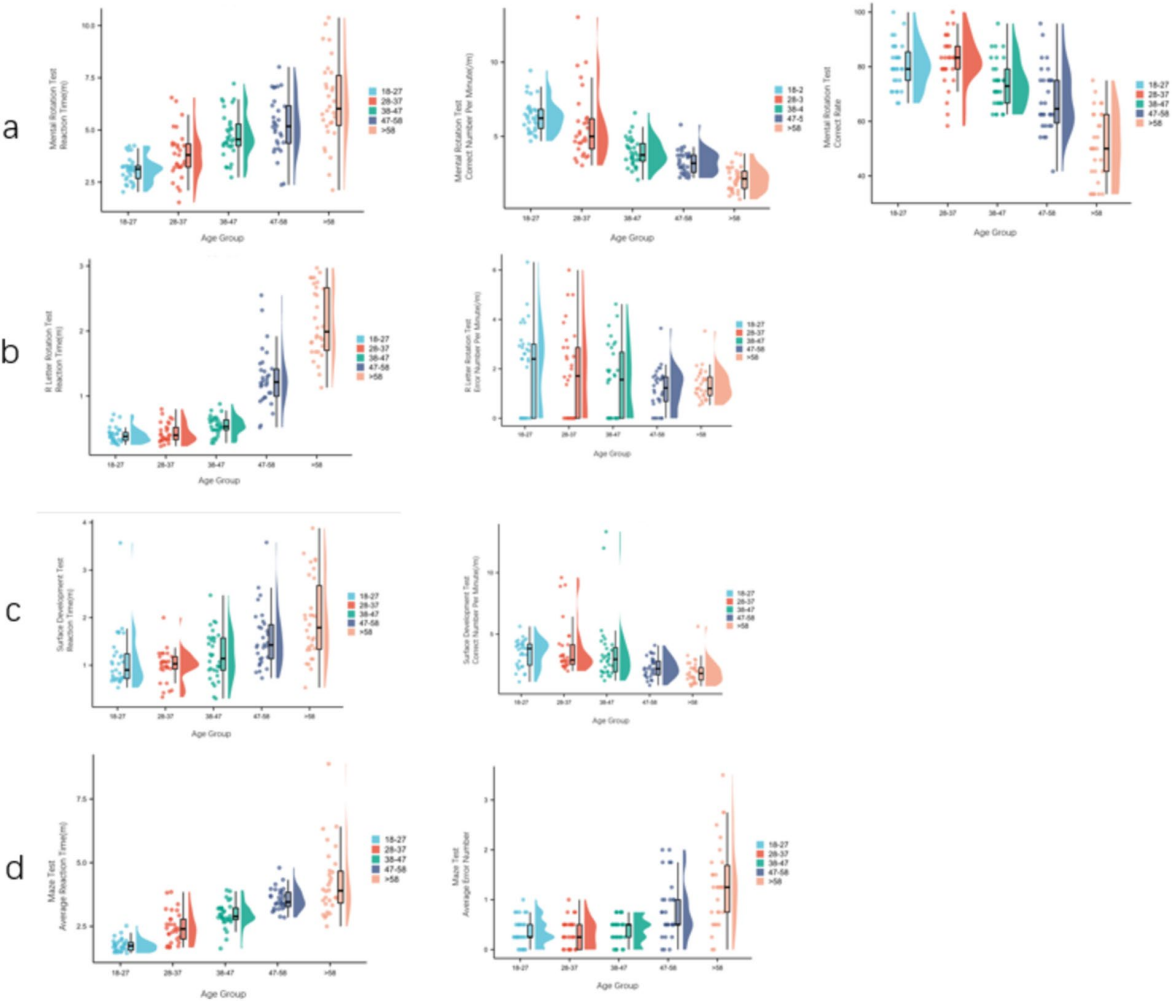
The development track of male’s visual ability and orientation ability in different age groups. **(A)** S-M mental rotation test scores (reaction time, correct number per unit time, correct rate) for each male age group. **(B)** R-letter rotation test scores (reaction time, number of errors per unit time) of each male age group. **(C)** Surface development test scores (reaction time, number of correct times per unit time) of each male age group. **(D)** Maze test scores (average reaction time, average number of errors) for each male age group.

The study described the development trajectory of the visual ability and orientation ability of 300 participants of the same gender and different age groups. SPSS 26.0 software was used for data analysis. Measurement data conforming to normal distribution were represented by (mean ± standard deviation), and independent sample *T*-test was used for comparison between the two groups. Single factor analysis of variance among multiple groups, LSD test was used for pound-for-pair comparison. Measurement data that did not conform to normal distribution were represented by M (P25–P75), and Wilcoxon rank sum test was used to compare the two groups. Kruskal–Wallis test of independent samples was used for comparison among multiple groups, and Bonferroni method was used for pairwise comparison of significance levels after pairwise comparison. *p* < 0.05 indicated statistical significance. In the S-M mental rotation test, the correct rate of males increased gradually from 18–27 years old to 28–37 years old, and then decreased steadily. The correct number of reaction time and unit time reached the peak at 18–27 years old, and then decreased gradually. Pairwise comparison between adjacent age groups showed that there was a significant difference in the correct number of unit time between >58 years old and 48–57 years old, and there was a significant difference in the correct rate between 38–47 years old and 28–37 years old (Figure 2A). In the R-letter rotation test, the reaction time gradually increased with age, and the number of errors per unit time gradually decreased. The pairwise comparison between adjacent age groups showed that there was a significant difference in reaction time between 48–57 years old and 38–47 years old (Figure 2B). In the surface development test, the reaction time gradually increased with age, and the number of correct times per unit time gradually decreased (Figure 2C). In the maze test, the average reaction time gradually increased with age, and increased rapidly after >58 years old. The pairwise comparison between adjacent age groups showed that there were significant differences in the average reaction time between 28–37 years old and 18–27 years old, 48–57 years old and 38–47 years old, and there was no significant difference in the average number of errors (Figure 2D).

**Figure 3:**
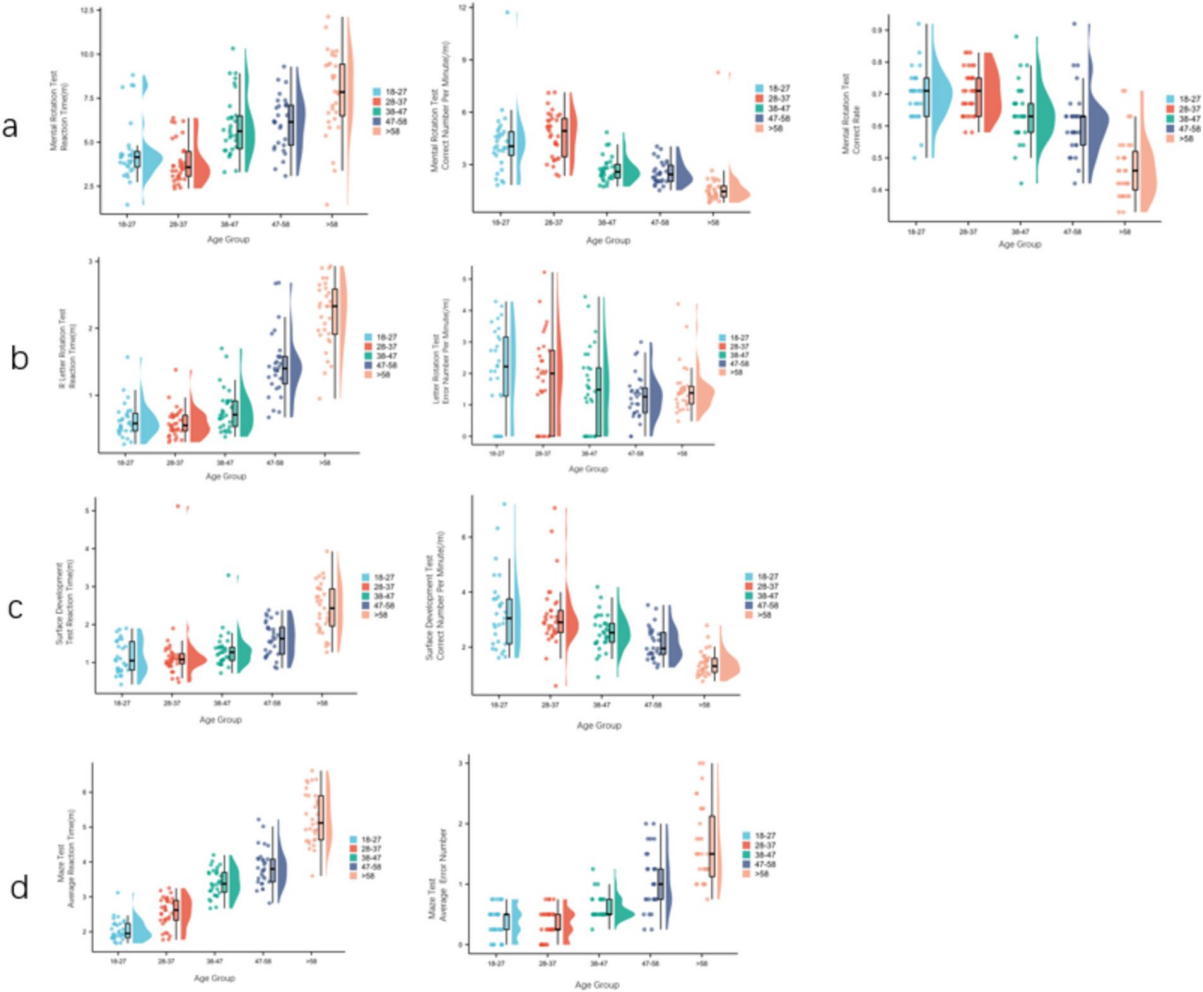
The development track of women’s visual ability and orientation ability in different age groups. **(A)** S-M mental rotation test scores of each female age group (reaction time, correct number per unit time, correct rate). **(B)** R-letter rotation test scores of each female age group (reaction time, wrong number per unit time). **(C)** Surface development test scores of each female age group (reaction time, correct number per unit time). **(D)** Maze test scores (average reaction time, average number of errors) for each female age group.

In the S-M mental rotation test, the correct number of unit time of women increased gradually from 18–27 years old to 28–37 years old, and then decreased rapidly. The response time of 28–37 years old was the least, and decreased rapidly after >58 years old. Pairwise comparison between adjacent age groups showed that there were significant differences in response time and correct number of unit time between 38–47 years old and 28–37 years old, and there were significant differences in correct rate between >58 years old and 48–57 years old (Figure 3A). In the R-letter rotation test, the 28–37 age group had the least reaction time, and the number of errors per unit time gradually decreased. There was no significant difference between the adjacent age groups (Figure 3B). In the surface development test, the reaction time gradually increased with age, and the number of correct times per unit time gradually decreased. The pairwise comparison between adjacent age groups showed that there were significant differences in reaction time and correct number per unit time between >58 years old and 48–57 years old (Figure 3C). The average reaction time gradually increased with age, and the average number of errors in the 28–37 age group was the least, and then increased rapidly. Pairwise comparison between adjacent age groups showed that there were significant differences in the average reaction time between 38–47 years old and 28–37 years old, >58 years old and 48–57 years old, and there were significant differences in the average number of errors between 48–57 years old and 38–47 years old (Figure 3D).

Among the 300 participants, we compared the development trajectories of different gender groups in the same age group to test the differences in spatial ability of different gender groups. In the 18–27 age group, men performed better than women in the S-M mental rotation test. In the R-letter rotation test, the reaction time of males was better than that of females. In the surface development test, there was no significant difference between men and women. In the maze test, the average reaction time of men was better than that of women (Table 1). In the age group of 28–37 years old, in the S-M mental rotation test, the correct number and correct rate of male unit time were better than those of female. In the R-letter rotation test, the reaction time of males was better than that of females. In the surface development test, there was no significant difference between men and women. There was no significant difference between men and women in the maze test (Table 2). In the 38–47 age group, men performed better than women in the S-M mental rotation test. In the R-letter test, male reaction time was better than female. In the surface development test, there was no significant difference between men and women. In the maze test, the average reaction time of men was better than that of women (Table 3). In the 48–57 age group, men performed better than women in the S-M mental rotation test. There was no significant difference between men and women in the R-letter rotation test. There was no significant difference between men and women in the surface development test. In the maze test, men performed better than women (Table 4). ≥ 58 years old, in the S-M mental rotation test, male performance is better than female. In the R letter test, there was no significant difference between men and women. In the surface development test, men performed better than women. In the maze test, the average reaction time of men was better than that of women (Table 5).

**Table 1:** Comparison of spatial ability between men and women aged 18–27 years.

	S-M mental rotation test			R letter rotation test		Maze test		Surface development test	
	Reaction time (m)	Correct number per minute (/m)	Correct rate	Reaction time (m)	Error number per minute (/m)	Average reaction time (m)	Average error number	Reaction time (m)	Correct number per minute (/m)
Male (*n* = 31)	3.13 (2.63,3.28)	6.23 (5.51,6.84)	0.79 (0.75,0.88)	0.38 (0.32,0.45)	2.40 (0.00,3.00)	1.73 (1.53,1.90)	0.25 (0.25,0.50)	0.90 (0.72,1.28)	3.83 (2.38,4.19)
Female (*n* = 29)	4.15 (3.54,4.55)	4.05 (3.33,4.92)	0.71 (0.63,0.75)	0.58 (0.47,0.74)	2.22 (0.64,3.25)	1.95 (1.83,2.23)	0.50 (0.25,0.50)	1.05 (0.79,1.56)	3.05 (2.11,3.79)
K-W H	4.327	6.662	6.666	4.256	0.075	4.020	0.353	0.903	1.317
*P*	<0.001	<0.001	<0.001	<0.001	0.940	<0.001	0.724	0.367	0.188

**Table 2:** Comparison of spatial ability between men and women aged 28–37 years.

	S-M mental rotation test			R letter rotation test		Maze test		Surface development test	
	Reaction time (m)	Correct number per minute (/m)	Correct rate	Reaction time (m)	Error number per minute (/m)	Average reaction time (m)	Average error number	Reaction time (m)	Correct number per minute (/m)
Male (*n* = 29)	3.80 (3.21,4.36)	5.00 (4.00,6.23)	0.83 (0.79,0.88)	0.40 (0.33,0.55)	1.71 (0.00,3.01)	2.40 (1.93,2.86)	0.25 (0.00,0.50)	1.03 (0.91,1.18)	2.90 (2.54,4.17)
Female (*n* = 33)	3.58 (3.00,4.50)	4.93 (3.41,5.72)	0.71 (0.63,0.75)	0.55 (0.44,0.70)	2.00 (0.00,2.76)	2.62 (2.32,2.92)	0.25 (0.25,0.50)	1.08 (0.95,1.25)	2.90 (2.51,3.46)
K-W H	0.254	6.6.760	6.765	3.029	0.072	1.122	0.441	1.327	0.798
*P*	0.800	<0.001	<0.001	0.002	0.943	0.262	0.659	0.184	0.425

**Table 3:** Comparison of spatial ability between men and women aged 38–47 years.

	S-M mental rotation test			R letter rotation test		Maze test		Surface development test	
	Reaction time (m)	Correct number per minute (/m)	Correct rate	Reaction time (m)	Error number per minute (/m)	Average reaction time (m)	Average error number	Reaction time (m)	Correct number per minute (/m)
Male (*n* = 32)	4.55 (4.21,5.35)	3.75 (3.27,4.62)	0.73 (0.67,0.79)	0.53 (0.48,0.65)	1.56 (0.00,2.80)	2.89 (2.75,3.22)	0.50 (0.25,0.50)	1.14 (0.89,1.58)	2.95 (1.93,4.06)
Female (*n* = 30)	5.63 (4.58,6.65)	2.59 (2.21,3.07)	0.63 (0.58,0.68)	0.71 (0.53,0.92)	1.48 (0.00,2.29)	3.38 (3.10,3.71)	0.50 (0.50,0.75)	1.27 (1.05,1.41)	2.53 (2.16,2.92)
K-W H	3.001	6.773	6.773	3.307	0.181	3.741	2.958	0.824	1.423
*P*	0.003	<0.001	<0.001	0.001	0.570	<0.001	0.003	0.410	0.155

**Table 4:** Comparison of spatial ability between men and women aged 48–57 years.

	S-M mental rotation Test			R letter rotation test		Maze test		Surface development test	
	Reaction time (m)	Correct number per minute (/m)	Correct rate	Reaction time (m)	Error number per minute (/m)	Average reaction time (m)	Average error number	Reaction time (m)	Correct number per minute (/m)
Male (*n* = 30)	5.18 (4.29,6.22)	3.20 (2.55,3.77)	0.65 (0.58,0.75)	1.22 (0.98,1.44)	1.23 (0.64,1.68)	3.46 (3.24,3.87)	0.50 (0.50,1.00)	1.43 (1.12,1.88)	2.18 (1.65,2.84)
Female (*n* = 29)	6.15 (4.78,7.14)	2.44 (2.04,3.03)	0.63 (0.54,0.63)	1.40 (1.14,1.58)	1.26 (0.74,1.57)	3.80 (3.43,4.09)	1.00 (0.75,1.38)	1.63 (1.22,1.99)	1.95 (1.67,2.55)
K-W H	2.100	6.606	6.718	1.661	0.084	2.192	2.134	0.902	0.796
*P*	0.036	<0.001	<0.001	0.097	0.933	0.028	0.033	0.367	0.426

**Table 5:** Comparison of spatial ability between men and women aged >58 years.

	S-M mental rotation test			R letter rotation test		Maze test		Surface development test	
	Reaction time (m)	Correct number Per Minute (/m)	Correct rate	Reaction time (m)	Error number per Minute (/m)	Average reaction time (m)	Average error number	Reaction time (m)	Correct number per minute (/m)
Male (*n* = 30)	6.02 (5.12,7.75)	2.15 (1.47,2.72)	0.50 (0.41,0.63)	1.99 (1.68,2.68)	1.21 (0.89,1.72)	3.90 (3.37,4.75)	1.25 (0.69,1.75)	1.79 (1.33,2.85)	1.81 (1.19,2.35)
Female (*n* = 31)	7.85 (6.33,9.47)	1.46 (1.14,1.80)	0.46 (0.38,0.54)	2.33 (1.90,2.60)	1.38 (1.02,1.61)	5.12 (4.62,5.90)	1.50 (1.00,2.25)	2.43 (1.92,2.95)	1.31 (1.07,1.61)
K-W H	2.770	6.715	6.718	0.592	0.556	3.903	1.876	2.438	2.273
*P*	0.006	<0.001	<0.001	0.554	0.579	<0.001	0.061	0.015	0.023

**Figure 4:**
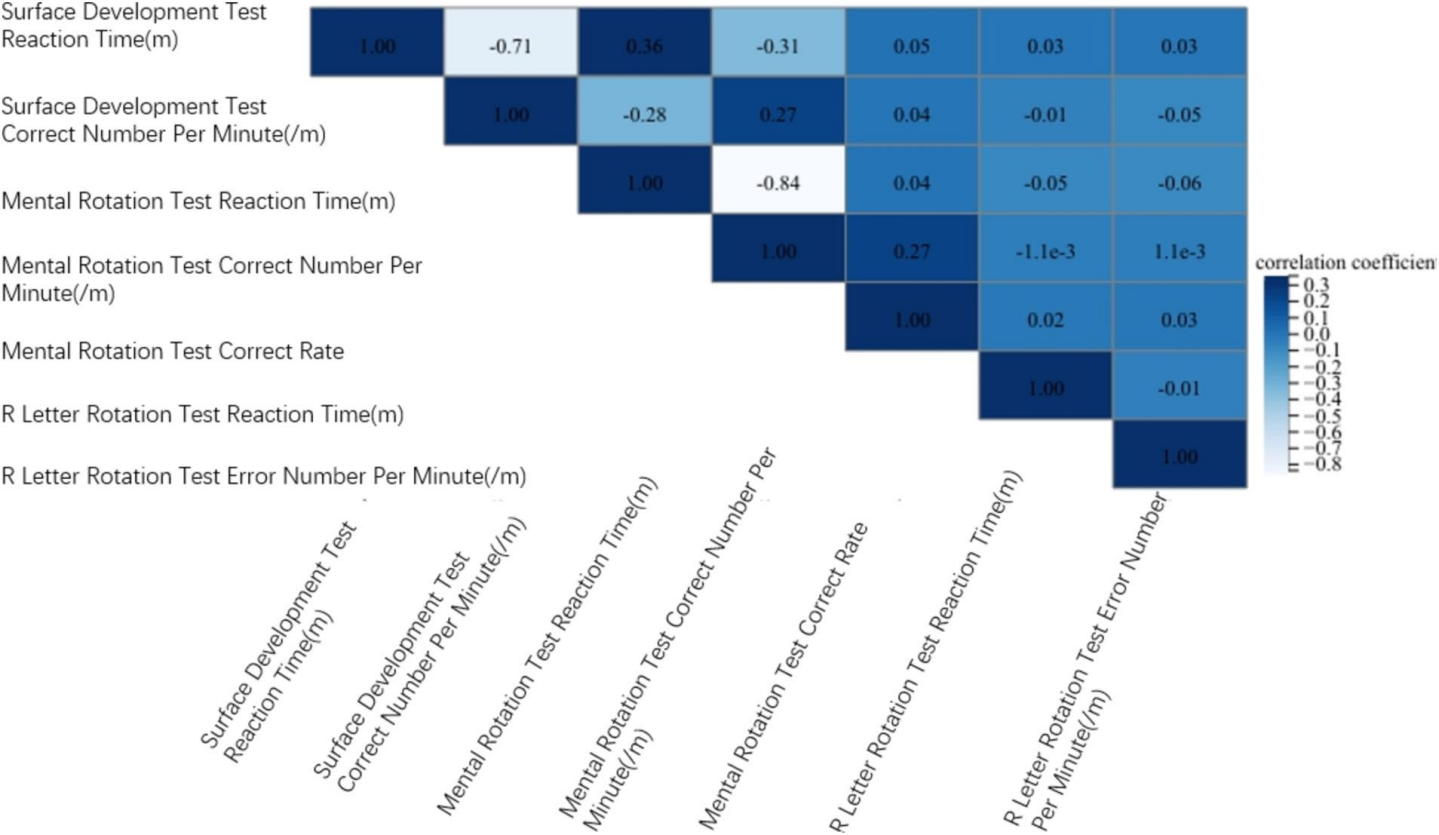
Correlation of visual ability test. Correlation analysis was performed on 143 participants S-M mental rotation test scores, R-letter mental rotation test scores, and surface development test scores.

In addition, 143 subjects were selected for visual ability test (S-M mental rotation test, R character rotation test, surface development test). The study found that although the S-M mental rotation test and the R-letter rotation test are both mental operation rotation ability, the R-letter rotation test is not related to the S-M mental rotation test, and is not related to the surface development test. The surface development test was weakly correlated with the S-M mental rotation test. There was no correlation between the number of errors per unit time in the R-letter rotation test and the number of correct per unit time in the surface development test (*r* = −0.045, *p* = 0.597). There was no correlation between the reaction time of R-letter rotation test and the reaction time of surface development test (*r* = 0.034, *p* = 0.688). There was a weak positive correlation between the correct number of unit time in S-M mental rotation test and the correct number of unit time in surface development test (*r* = 0.273, *p* = 0.001). There was a weak positive correlation between the reaction time of S-M mental rotation test and the reaction time of surface development test (*r* = 0.357, *p* < 0.001) (Figure 4).

**Figure 5:**
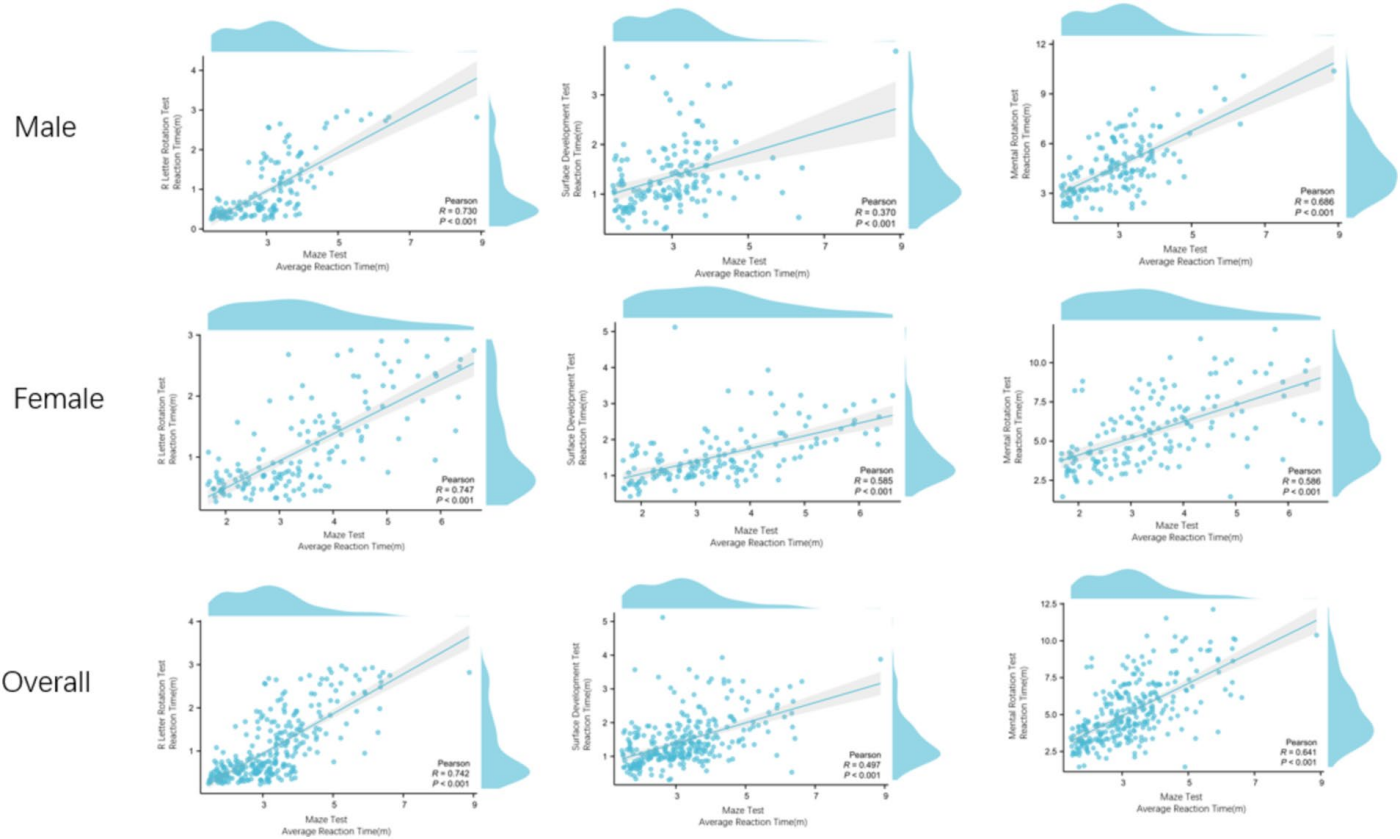
The relationship between visual ability and spatial orientation ability in reaction time. Regardless of gender, visual ability and orientation ability are positively correlated.

**Figure 6:**
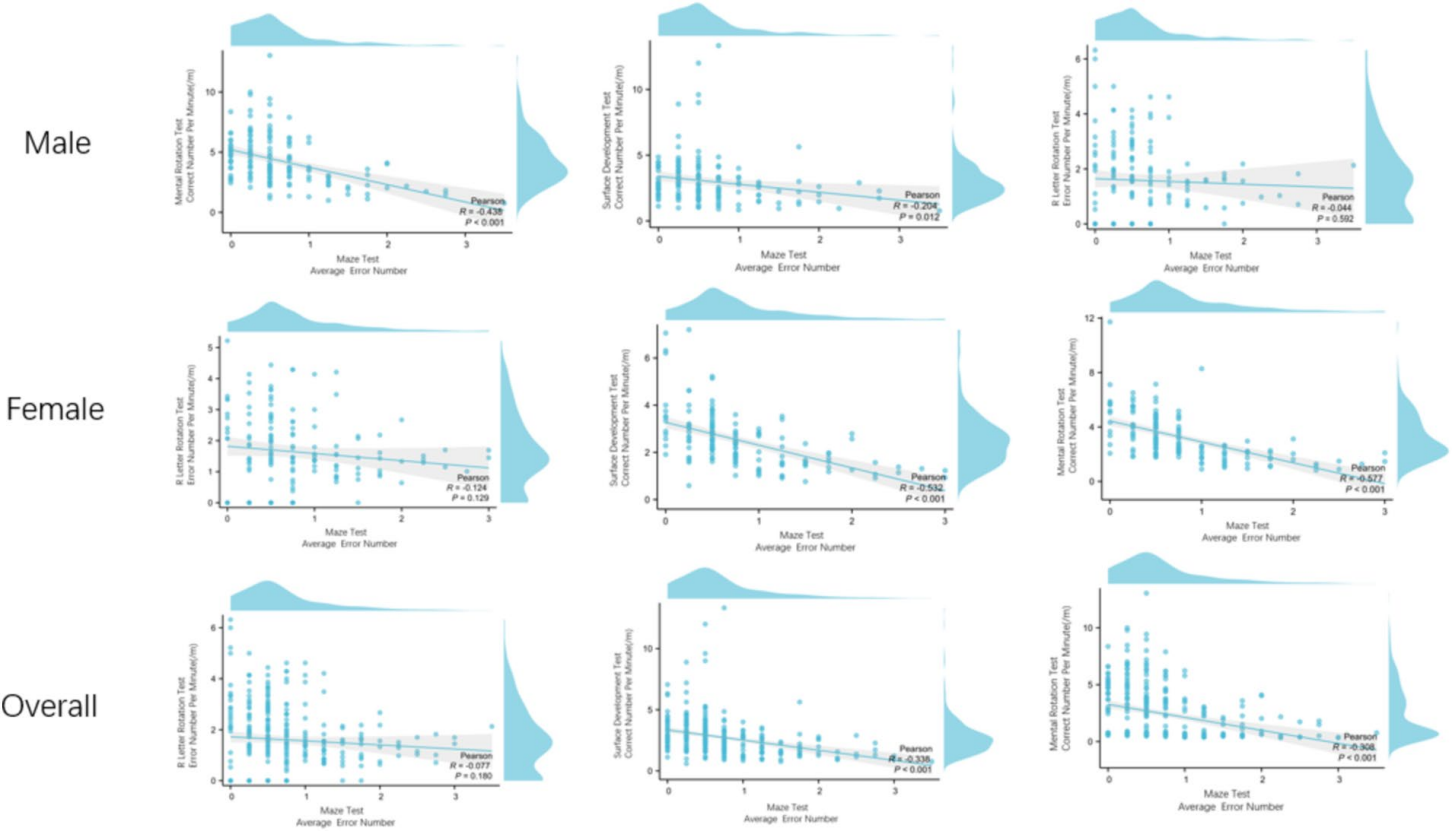
The relationship between the correct number per unit time of the visual test and the average number of errors in the directional ability test. Regardless of gender, the correct number per unit time of S-M mental rotation test and surface development test was negatively correlated with the average number of errors in the maze test, and the number of errors per unit time of R letter rotation was not correlated with the average number of errors in the maze test.

We analyzed the correlation between spatial visualization ability and spatial orientation ability of 300 subjects. In terms of reaction time, the S-M mental rotation test, surface development test and R-letter rotation test of different gender groups were positively correlated with the maze test. The overall results are the same as above (Figure 5). Male S-M mental rotation test unit time correct number and surface development test unit time correct number were negatively correlated with the average number of errors in the maze test (*r* = −0.438, *p* < 0.001; *r* = −0.204, *p* = 0.012). There was no correlation between the number of errors per unit time in the R-letter rotation test and the average number of errors in the maze test (*r* = −0.044, *p* = 0.592). Female and overall results are the same as above (Figure 6).

## Discussion

Spatial cognitive ability plays a central role in spatial information processing, perception and action. It is used to improve the adaptability and effectiveness of perception, cognitive processing and motor action. The integrated analysis of spatial ability can learn more information than individual analysis. This study integrates visual ability and spatial orientation ability to explore the age effect of different gender groups, the correlation of cognitive ability and the independence of different spatial ability tests.

Several studies have shown that the cognitive ability of adults will gradually decline with age after reaching the best level (Malanchini et al., 2020), which is different from the results of this study. The results of male spatial visualization test gradually decreased with age, but in the S-M mental rotation test, the correct rate of 28–37 years old reached the peak, and the correct number of unit time in the surface development test increased from 28–37 years old to 38–47 years old. The average reaction time in the spatial orientation test increased with age, the average number of errors in the adjacent age groups was consistent, and the average number of errors in the ≥58 years old increased rapidly. This is consistent with the results of Liu et al. (2011) that there are significant differences in the accuracy of young and old people in completing orientation tasks. The study also did not find significant differences in performance between the two younger age groups (18–30 years old vs. 31–45 years old), which is also consistent with our findings. In the spatial visualization ability, women are different from men. In the mental rotation and R character rotation experiments, the level of 28–37 years old is higher than that of other age groups, and the spatial orientation ability decreases with age. The cognitive level of women aged 28–37 is higher than that of other age groups. This spatial ability may be promoted by experience and the best estrogen-androgen balance, so this phenomenon may be affected by the combined effects of hormones, experience and other factors. The study found that professional knowledge and experience factors delayed the effect of age on the reduction of spatial ability (Parsons, 2004; Morrow et al., 2001). The study proposed the importance of empirical factors in the development of spatial skills (Berry, 1966). The factors of individual differences in spatial test scores are divided into four categories: environment, genetics, hormones and neurology. The assessment of all cognitive tasks has age-related decline. High estrogen levels are associated with the use of location memory, and low estrogen levels are associated with the use of response memory. Estrogen levels enable women to use different memory systems to solve tasks (Janowsky et al., 1998; McGee, 1979) found that normal spatial ability requires at least the lowest androgen level. The age development trajectory of the spatial ability of men and women, the development trend of men and women on the S-M mental rotation test, the R letter rotation test, the surface development test, and the maze test are similar, and the spatial ability decreases with age. Considering the age-related changes in cognitive ability discussed above, even if you have certain work experience or are affected by hormones, human cognitive ability will decline with age. The age effect of spatial ability in different gender groups is different. With the increase of age, the experience factor has little effect on men, but the effect of experience factor on women is more obvious. In career selection, specific analysis should be made for different gender and age groups.

Men performed better than women in all spatial tests (Klencklen et al., 2012; Malanchini et al., 2020). Many scholars have discussed the gender differences in spatial visualization (Voyer et al., 2004). Women lag behind in tasks with spatial factors, and the differences between men and women increase with age. This is different from the results of this study. The visual ability is not synchronized between the different categories of abilities between men and women. In the surface development test, in the ≥58 age group, men performed better than women, and there was no statistical difference between men and women in other age groups, which was contrary to the results of the R-character rotation test. This may be due to the lack of sample size, and it is necessary to increase the sample size for testing. Compared with men and women in the same age group, S-M mental rotation test and R letter mental rotation test showed that men performed better than women. In the maze test, there was no difference between men and women in the 28–37 age group, and there was no significant difference between men and women in the ≥58 age group. In other age groups, men perform better than women. Men usually perform better than women in the S-M mental rotation test and spatial orientation task, while women perform better than men in the object location memory test. The analysis of the characteristics or processes of visual cognition provides accurate information on the types of tasks that different gender groups may exhibit at different levels of performance (Cohen, 1976). Skills such as visualization and perception can explain these differences (Cimadevilla and Piccardi, 2020).

An important finding of this study is that the R-letter rotation test and the S-M mental rotation test are independent. The S-M mental rotation test cube is rotated along the Z axis, compared to the fixed cube to judge (object-object representation system), while the rotation of the R letter is rotated through the plane, only the tilt angle of the R letter is changed (self-object representation system). The two test studies compared different rotation abilities. Although the two tasks are logically equivalent, the results showed by the subjects were not significantly correlated. Although both tests operate object rotation through psychological imagination, the way of rotation is different from the reference system (Frick, 2019; Uttal et al., 2013), and the two are independent conclusions. These results are consistent with behavioral and neuroscientific evidence (Bryant and Tversky, 1999; Zacks et al., 1999), because there is a separation between the object-object representation system and the self-object representation system. Although the use frequency of the two rotation methods is the same, the influence of different rotation dimensions on the structure of spatial ability is not clear, and the structure and influence of mental rotation need to be further explored.

Importantly, this study expands on the basis of previous studies from three aspects. First, the study found that the R-letter rotation test and the S-M mental rotation test were independent, and the different spatial tests assessed only partial abilities. In addition, different reference frames and rotation dimensions lead to different representations of an object’s rotation. The structure of mental rotation ability is complex and its mechanism needs to be further studied. This provides the importance of reference frame selection for subsequent research and opens up new research directions on whether different rotation dimensions affect the structure of the ability to operate rotation. Secondly, the study examined the developmental trajectory of spatial cognitive ability in adult life cycle of both sexes and age. Age is an important factor affecting spatial ability (Gyselinck et al., 2013; Muffato et al., 2024). Both male and female spatial visualization ability and spatial orientation ability show age-related degradation. Age trends are similar for men and women, but women reach the peak of spatial ability in early adulthood. Experience factors play an important role (Schug, 2024), and the effect on women is more obvious, and the effect on men is not as strong as age factors. We improve women’s spatial ability through relevant exercises, which plays an important role in career selection for different gender groups. Third, men’s advantage over women varies across different tests of spatial ability. On tests of surface development, men do not outperform women until late adulthood or even old age. Sufficient practice will increase the advantage of spatial ability, which may encourage further participation in space-related tasks. In old age, men outperform women on all spatial tests, which may be the result of hormonal influences or gender-dependent brain polymorphisms. However, the evidence for this effect is mixed and mixed in the literature (Herlitz et al., 2013). The complex structure of age effects, dissynchrony between tests and mental rotation ability in different gender groups does not exist by coincidence, and further study of the causal relationship between these factors is needed.

Based on virtual reality technology, this study has made a series of achievements in evaluating spatial ability of normal people, but there are still many problems to be explored. First of all, in terms of training method optimization, since it has been clear that the development track of spatial ability is related to age and gender, training tasks targeting different dimensions such as mental rotation, surface development and spatial orientation can be designed in the future, so as to explore the most effective training methods for specific spatial ability and the effect differences of different methods in different genders and age groups.

Secondly, the application prospect of virtual reality technology in space ability training is broad. With its realistic 3D environment, interactivity and real-time feedback, it is expected to develop more personalized training programs in the future.

Finally, technological developments have promoted interdisciplinary research, integrating theoretical approaches from neuroscience, psychology, and computer science to analyze the nature of spatial ability from multiple perspectives. For example, fMRI was used to monitor changes in brain activity and reveal the neural mechanisms behind the tasks as well as age and gender differences. In short, the field of spatial ability research is unknown, and this study lays the foundation for subsequent research. It is expected that by exploring the above new questions and hypotheses in depth, the mystery can be further revealed, and the level of human spatial cognition and related practical applications can be improved.

## Data Availability

The raw data supporting the conclusions of this article will be made available by the authors, without undue reservation.
